# How Do Polyphenol-Rich Foods Prevent Oxidative Stress and Maintain Gut Health?

**DOI:** 10.3390/microorganisms12081570

**Published:** 2024-07-31

**Authors:** Samir Jawhara

**Affiliations:** 1 Centre National de la Recherche Scientifique, UMR 8576—UGSF—Unité de Glycobiologie Structurale et Fonctionnelle, F-59000 Lille, France; samir.jawhara@univ-lille.fr; Tel.: +33-(0)3-20-62-35-46; 2 Institut National de la Santé et de la Recherche Médicale U1285, University of Lille, F-59000 Lille, France; 3 Medicine Faculty, University of Lille, F-59000 Lille, France

**Keywords:** polyphenol, inflammatory bowel disease, reactive oxygen species generation, oxidative stress, fungal growth, microbiota, healthy diet

## Abstract

Inflammatory bowel disease (IBD), which includes Crohn’s disease and ulcerative colitis, involves chronic inflammatory disorders of the digestive tract. Oxidative stress, associated with increased reactive oxygen species generation, is a major risk factor for IBD pathogenesis. Industrialized lifestyles expose us to a variety of factors that contribute to deteriorating gut health, especially for IBD patients. Many alternative therapeutic strategies have been developed against oxidative stress along with conventional therapy to alleviate IBD pathogenesis. Polyphenol-rich foods have attracted growing interest from scientists due to their antioxidant properties. Polyphenols are natural compounds found in plants, fruits, vegetables, and nuts that exhibit antioxidant properties and protect the body from oxidative damage. This review presents an overview of polyphenol benefits and describes the different types of polyphenols. It also discusses polyphenols’ role in inhibiting oxidative stress and fungal growth prevention. Overall, this review highlights how a healthy and balanced diet and avoiding the industrialized lifestyles of our modern society can minimize oxidative stress damage and protect against pathogen infections. It also highlights how polyphenol-rich foods play an important role in protecting against oxidative stress and fungal growth.

## 1. Introduction

Inflammatory bowel disease (IBD) includes Crohn’s disease (CD) and ulcerative colitis, two chronic inflammatory diseases of the gastrointestinal tract (GI) with extremely complex etiologies [1,2]. The etiology of IBD is not yet fully understood, but it is believed to involve a combination of genetic, environmental, and immune system factors [1,2]. Genetic predisposition, environmental factors, gut dysbiosis, and an overactive immune response are all thought to play a role in IBD development and progression [3,4]. IBD has increased significantly in the past few decades, affecting 3.1 million adults in the U.S. and 2.2 million in Europe, as well as an increase in Asian populations [5,6,7]. The rising incidence of IBD in industrialized countries suggests a possible link between the disease and modern lifestyle factors such as diet, stress, and exposure to environmental toxins. Additionally, changes in gut microbiota composition due to the Westernization of diets and reduced exposure to beneficial bacteria may also contribute to the development and progression of IBD [8,9,10].

In recent years, research has increasingly focused on the gut microbiota’s role in IBD [11,12]. The breakdown of host–microbial mutualism is probably what defines IBD development as a definitive change in the normal gut microbiota [13].

The gut microbiota has been consistently altered in IBD patients, particularly by Firmicute reduction. *Bacteroides* species may have been spatially reorganized in IBD patients, with *Bacteroides fragilis* occupying an increased proportion of the bacterial mass [14]. A shift in the balance between beneficial and harmful bacteria is observed in IBD pathogenesis, marked by a decline in *Firmicutes* and a spatial redistribution of *Bacteroides* species.

Among *Firmicutes* species, *Faecalibacterium prausnitzii* (*F. prausnitzii*) decreases significantly in patients with CD, particularly those with ileal CD, suggesting that *F. prausnitzii* is an anti-inflammatory bacterium. In addition, it is likely that its decrease in adult CD patients is a result of inflammation caused by the disease. A pediatric cohort has shown an increase of *F. prausnitzii*, suggesting a more dynamic role for the species [15].

In addition to the reduction in *Firmicutes* and the spatial reorganization of *Bacteroides* species, IBD is linked to increased members of the Proteobacteria phylum, which have been identified as key players. The Proteobacteria phylum is associated with the production of endotoxins, which are molecules that can stimulate an inflammatory response. This inflammation then triggers the other components of the immune system, leading to further inflammation and the development of IBD [16].

The increase in *E. coli*, a member of the Proteobacteria phylum, in patients with IBD, especially those with ileal CD, suggests its potential role in the development and progression of the disease. The immune response triggered by *E. coli* endotoxins in IBD contributes to chronic inflammation, tissue damage, and immune cell recruitment [17].

During mucosal inflammation, inflammatory cytokines activate NADPH oxidase (NOX) and inducible nitric oxide synthase (iNOS), leading to the release of superoxide from intestinal epithelial cells, neutrophils, and macrophages [18]. Excessive levels of reactive oxygen species (ROS) referring to oxidative stress can damage proteins in the cytoskeleton, contributing to inflammation and increasing the permeability of tight junctions in intestinal walls. This disruption of the intestinal epithelial barrier ultimately results in further mucosal inflammatory responses [19]. There is evidence that inflammation of the gut caused by oxidative stress is the precursor to the onset of IBD in humans [19].

Microvascular networks that surround epithelial cells can attract inflammatory mediators, leading to more tissue damage and an escalation of intestinal inflammation caused by circulating inflammatory mediators [20]. Various morphological lesions have been associated with intestinal inflammation, including the loss of goblet cells, decreased mucus production, ulceration, and hyperplasia of colonic crypt cells [21,22]. These morphological epithelial lesions contribute to reduced intestinal barrier function, allowing pathogenic bacteria and toxins into the circulation and causing systemic inflammation that leads to further tissue damage.

## 2. An Overview of Adverse Factors That Trigger Oxidative Stress, with a Special Focus on ROS Generation in IBD

**Figure 1:**
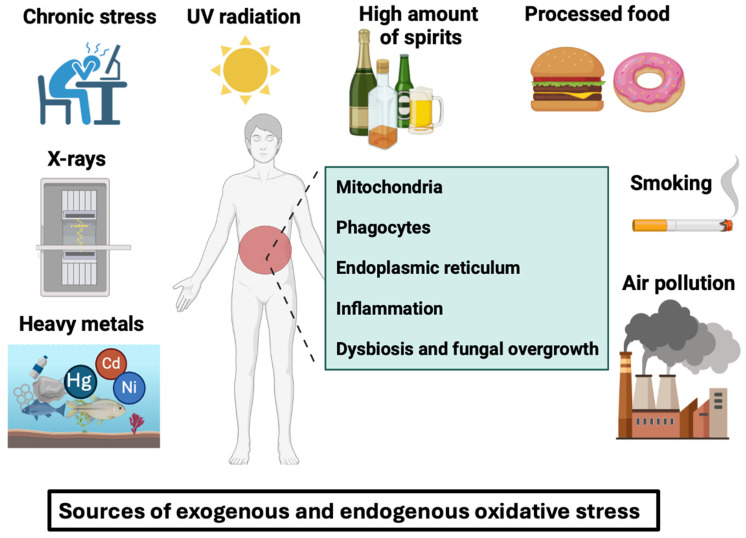
Oxidative stress can be defined as an imbalance caused by excessive reactive oxygen species production by cells that exceeds the body’s capacity to neutralize them. Free radicals and peroxides damage intracellular structures such as proteins, lipids, and DNA and disrupt intrinsic cell mechanisms. These free radicals can be induced by exogenous factors such as smoking, air pollution, processed foods, chronic stress, UV radiation, X-rays, heavy metals (e.g., nickel, cobalt, and mercury) or by endogenous factors like intracellular mitochondria, phagocytes, endoplasmic reticulum, inflammation, gut dysbiosis, and fungal overgrowth in the gut.

Our current industrialized lifestyle exposes us to a variety of exogenous unhealthy factors (smoking, processed food, hydrogenated oils, chronic stress, alcohol, air pollution, heavy metals, and ultraviolet light) and endogenous conditions (mitochondria and phagocyte NADPH oxidases) that can damage our cells and deteriorate our digestive tract, especially for IBD patients. These unhealthy factors can lead to chronic inflammation and oxidative stress, further exacerbating IBD pathogenesis and progression (Figure 1).

**Smoking** is one of the most significant sources of ROS, which can have adverse effects on the GI tract [23]. The smoking substance list includes aldehydes, quinones, epoxides, nitric oxides, and several other compounds which are sources of ROS production [24]. The most common GI disorders include CD, reflux disease, cancers of the esophagus, and many more [24].

In terms of **processed food**, a clinical study showed that antioxidant enzyme activities such as catalase (CAT) and superoxide dismutase (SOD) are low in ultra-processed food consumers. However, ROS generation and myeloperoxidase activity are higher in these consumers [25]. Additionally, the study conducted by Narula et al. showed that individuals who consume ultra-processed foods (soft drinks, refined sweetened foods, salty snacks, and processed meats) are more likely to develop IBD [26]. The intake of white meat, red meat, dairy products, starches, fruit, vegetables, and legumes was not associated with incidents of IBD [26]. Of note, high consumption of red meat has been linked to a higher incidence of colon cancer [27]. The high iron content of red meat may catalyze ROS formation in the colon during digestion [27]. 

**Trans fatty acids** are found in partially hydrogenated oils, which are commonly used in processed foods such as fried and baked goods, margarine, and snack foods. These unhealthy fats have been linked to the presence of acrylamide in food [28]. These trans fatty acids increased phosphorylation of NF-κB and ROS generation in human aortic endothelial cells [28].

**Alcohol**, and especially distilled spirits, in high amounts can damage the mucosal layer of the GI tract [29]. Additionally, alcohol can disrupt the balance of beneficial gut bacteria, further compromising the health of the GI tract. ROS generation from ethanol can contribute to liver diseases caused by alcohol consumption [30,31,32]. Of note, a breakdown of alcohol in the liver by cytochrome reductase CYP2E1 forms acetaldehyde during excessive alcohol consumption. This enzyme CYP2E1 can transfer electrons to oxygen to form a superoxide (O_2_^.−^) radical or catalyze lipid peroxidation that results in ROS generation [30,31,32].

**Chronic stress** is another factor linked to increased oxidative stress levels in our modern society [33,34]. Many approaches to dealing with stress situations can help minimize oxidative stress in individuals with chronic stress. It is important for these individuals to know how to handle difficult and stressful situations by choosing the most effective stress-handling techniques for themselves. These techniques include analyzing and planning future tasks, socializing, meeting with friends, explaining the situation, and finding solutions to problems [35,36,37]. It is also worthwhile to focus on self-care activities such as engaging in hobbies and exercising meditation and relaxation techniques.

**Air pollution** is considered one of the world’s leading environmental health threats [38,39]. Various pollutants are emitted by industrial processes, including particulate matter (PM), nitrogen oxides (NO_2_), sulfur dioxide (SO_2_), and volatile organic compounds, which can have adverse effects on both the environment and health [38,39]. The study of Jin et al. showed that PM penetration into damaged skin leads to inflammation and adverse effects on the skin [40]. Experimental studies conducted on animals have shown that the adverse effects of ROS activity on rodent respiratory systems depend on particle size and emission sources [41].

In terms of **heavy metals**, some individuals are exposed to redox-inert elements such as cadmium and arsenic which are toxic at low concentrations [42]. These individuals living in contaminated areas are exposed to these elements through a variety of natural sources, such as air pollution and contaminated drinking water and food [42].

It is well known that human skin is susceptible to constant exposure to **ultraviolet** (**UV) light** [43]. Exposure to the sun stimulates melanin synthesis, which results in post-inflammatory hyperpigmentation of the skin [44]. Excessive sunlight exposure generates ROS from epidermis melanocytes, which adversely affects these cells. An excessive increase in ROS generation could disrupt homeostasis, leading to the malignant transformation of these cells [43,44].

Among the endogenous sources of cellular ROS generation, **mitochondria and phagocyte NADPH oxidases** are the two most important sources of cellular ROS generation [45]. ROS are produced in mitochondria by the oxidation of metabolic intermediates in the electron transport chain (ETC). However, this process is tightly regulated to prevent oxidative damage to cells [46]. Mitochondrial ROS are produced via ETC in the form of superoxide, with complex I being the most common source of ROS in mitochondria [46]. Phagocyte NADPH oxidase (e.g., NOX2) is responsible for the generation of large amounts of ROS in phagosomes, which function as a direct or indirect mechanism of killing ingested microbes [45,47].

In addition to mitochondrial and phagocyte NADPH oxidases, ROS can also be generated by inflammation and gut dysbiosis (Figure 1). A variety of enteric pathogens are able to cause inflammation by stimulating the production of proinflammatory cytokines, which are further responsible for oxidative stress production. The damage caused by oxidative stress is aggravated by leukocyte activation in chronic intestinal disorders [48]. These factors result in excessive ROS production, which exceeds the antioxidant defenses and perpetuates or worsens inflammation in the mucosa. ROS molecules generated by unstable types of oxygen, such as superoxide ions, hydrogen peroxides, and hydroxyl radicals, are the major pro-oxidant molecules involved in the oxidative reaction [49]. These highly reactive molecules contribute to oxidative damage in the mucosa, exacerbating inflammation in chronic intestinal disorders.

## 3. Overview of Oxidative Stress Inhibition Approaches in IBD

**Figure 2:**
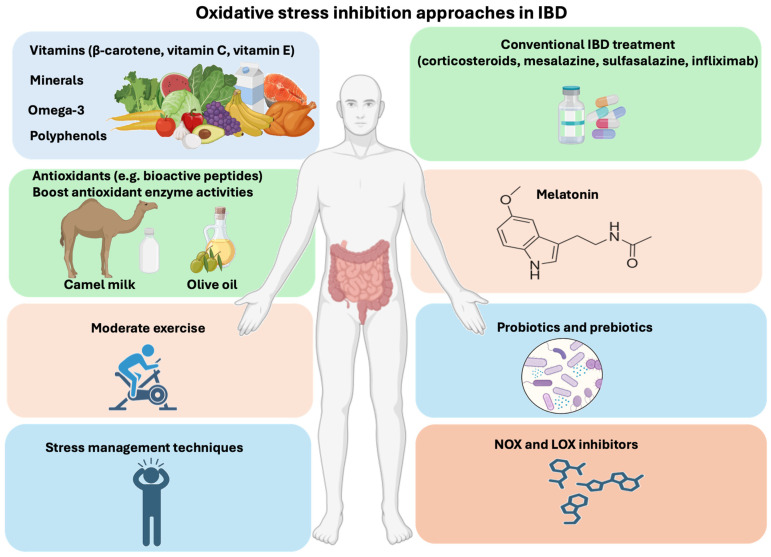
Oxidative stress inhibitors for IBD. Along with conventional therapy, many alternative therapeutic strategies have been developed to alleviate IBD pathogenesis. Various options for reducing oxidative stress in IBD include antioxidants such as melatonin or LOX and NOX inhibitors. In addition, lifestyle modifications by consuming foods rich in fruits and vegetables and medicinal plants rich in β-carotene (pro-vitamin A), vitamin C, vitamin E, minerals, omega-3 polyunsaturated fatty acids, and polyphenols and engaging in regular exercise in combination with foods abundant in probiotics and prebiotics, can reduce oxidative stress and inflammation. Some foods (e.g., olive oil) increase antioxidant enzyme activities (CAT, SOD, and GPx). In addition, camel milk contains lactoferrin and antioxidants (bioactive peptides and vitamin C), which protect against oxidative stress in IBD patients.

The conventional therapeutic approach to treating IBD consists of anti-inflammatory medicines such as corticosteroids, mesalazine, sulfasalazine, and infliximab for quick relief from IBD discomfort [50,51]. In parallel, many complementary therapeutic approaches, including some in clinical trials, are used to alleviate oxidative stress in IBD patients (Figure 2). One of these approaches, a new class of **NOX inhibitors**, shows potential anti-ROS properties. In addition to NOX inhibitors, LOXs (lipoxygenases) are a group of enzymes involved in the metabolism of arachidonic acid and other polyunsaturated fatty acids in cells [52]. Overactivation of LOXs generates ROS in cells. However, **LOX inhibitors** protect against inflammatory diseases and prevent ROS generation (Figure 2) [53].

**Melatonin**, a hormone synthesized in the pineal gland that regulates the sleep–wake cycle, can also block oxidative stress through its ability to cross physiological barriers, such as the mitochondrial membrane [54]. Melatonin’s antioxidant activity plays a protective role in the early and advanced stages of several diseases that involve ROS metabolites, including IBD [54,55,56]. 

The consumption of a healthy diet, including fruits and vegetables as well as medicinal plants abundant in **β-carotene (pro-vitamin A), vitamin C, vitamin E, minerals, omega-3 polyunsaturated fatty acids, and polyphenols** can reduce oxidative stress [57,58]. It is worth noting that some foods (e.g., olive oil) increase the amount of antioxidant enzymes such as CAT, SOD, and glutathione peroxidase (GPx) that can also prevent ROS production in the body [59,60]. 

In addition to fruits, vegetables, and medicinal plants that offer a variety of benefits to patients with IBD, **camel milk** provides an abundant source of minerals, vitamins, insulin, lactoferrin, and antioxidants (e.g., bioactive peptides) that have potential as a nutritional supplement for IBD patients [61,62,63]. With its antioxidant and anti-inflammatory properties, and its low content of sugar (lactose), protein (casein), saturated fat, and cholesterol, camel milk makes a valuable option for replenishing and improving IBD patients’ immune responses [64]. 

**Moderate exercise** and **stress management techniques** improve quality of life, reduce chronic inflammation, and enhance the immune response [37,65]. The study of Agarwal et al. showed that exercise training significantly reduced ROS production in animals’ hearts, as well as induced upregulation of antioxidant enzymes, which promoted a low-redox environment [66].

**Probiotics**, especially lactic acid bacteria, are living microorganisms involved in the balance of the microbiota in the gut [67,68]. There are a variety of foods rich in lactic acid bacteria, such as yogurt, kefir, sauerkraut, and kimchi [69,70,71]. Many beneficial biological effects are reported for probiotics belonging to the genera *Lactobacillus* and *Bifidobacterium*, particularly *Lactococcus lactis*, which has an antioxidative mechanism that involves scavenging oxidants, chelating metal ions, and preventing ROS formation [67,72].

**Prebiotics** are found in a variety of foods (fruits and vegetables) like fructo-oligosaccharides, which are non-digestible carbohydrates [73,74]. Prebiotics help the growth of beneficial bacteria in the gut, maintaining a balanced and healthy gut microbiota. The fermentation of prebiotics by *Bifidobacteria* has been linked to an improvement in oxidative parameters, which reduces free radicals. This effect can be explained by the production of hydrogen gas in the large intestine, which stimulates the growth of lactic acid bacteria [75,76].

## 4. Polyphenol-Rich Foods

**Figure 3:**
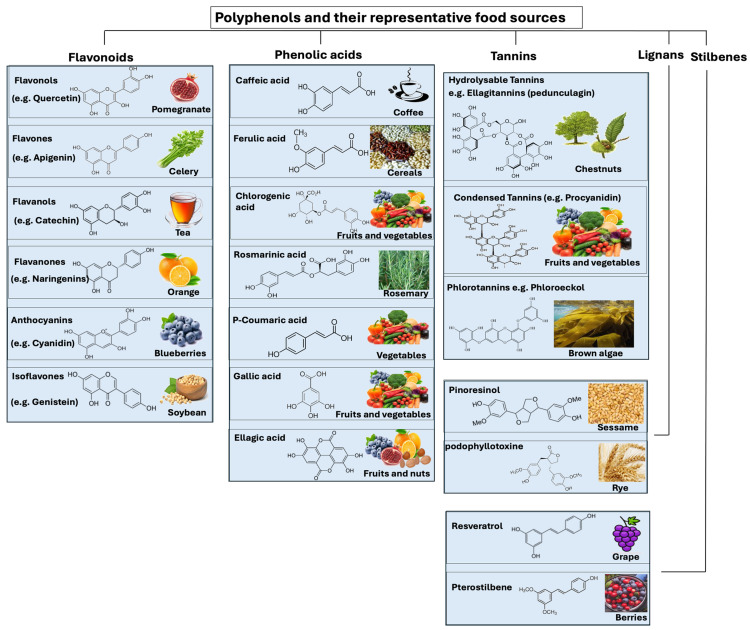
Polyphenols and their representative food sources. Polyphenols are classified into different groups such as flavonoids, phenolic acids, tannins, stilbenes, and lignans.

During the last decade, there has been a growing interest in polyphenol-rich foods and their potential role as antioxidants in preventing oxidative stress. Polyphenols are natural compounds found in plants, fruits, vegetables, and nuts (Figure 3). The antioxidant properties of these polyphenols include their ability to scavenge free radicals. Polyphenols protect plants from ultraviolet radiation and pathogens. Depending on how many phenol-rings the polyphenols contain, in addition to the structural elements that connect these rings together, polyphenols are classified into different groups such as flavonoids, phenolic acids, tannins, stilbenes, and lignans (Figure 3). 

**Flavonoids** are polyphenolic compounds found in plants, fruits, vegetables, and leaves. Several studies have demonstrated that diet-related polyphenols may protect against lifestyle-related diseases such as metabolic syndrome, atherosclerosis, coronary heart disease, and osteoporosis [77,78,79,80]. Two benzene rings are found in a flavonoid, which are linked to one another by a heterocyclic ring containing oxygen, which gives the flavonoid its structure [81]. In the case of flavonoids, the subclasses of flavonoid compounds include flavanols, flavanones, flavones, isoflavones, flavonols, and anthocyanidins [82]. Foods that contain flavonoids include fruits, vegetables, legumes, tea, dark chocolate, etc. Some foods have more flavonoids in specific parts, such as fruit peels. Flavonoid-containing foods can vary across countries based on culinary habits [83]. During digestion, flavonoids are metabolized through the intestine, and their metabolites are transported to the liver for further metabolic processing [81,84]. By entering the enterohepatic circulation through bile excretion, liver metabolites can be transported to target cells, metabolized by the microbiota into aglycones, or excreted through urine and feces [84]. Flavonoid metabolites that pass through the intestine but are not absorbed can be degraded by gut microbiota and reabsorbed [84]. In general, flavonoids have a low bioavailability, depending on their subclass [85]. Several approaches have been used to improve flavonoids’ bioavailability, including inhibition of enzymes that limit their availability, changes in the food matrix composition, and enhanced dissolution rates [86,87].

**Phenolic acids** are categorized into two groups belonging to benzoic acid and cinnamic acid hydroxy derivatives. They are bound in forms such as esters, amides, and glycosides. Lignin is one of the most common compounds containing hydroxybenzoic acids in their cell wall fractions. Gallic, syringic, p-hydroxybenzoic, and vanillic acids are well-known hydroxybenzoic acids [86]. The hydroxycinnamic acids (caffeic acid, ferulic acid, coumaric acid, chlorogenic acid, and isoferulic acid) are found in all parts of plants. They are most concentrated in ripe fruits and vegetables. Coffee contains high concentrations of chlorogenic acids, which are hydroxycinnamic acids obtained from the combination of caffeic and quinic acids. Considering phenolic acids’ role in diets, it has been shown that drinking coffee regularly reduces type 2 diabetes risk [88]. Additionally, the chlorogenic acid in coffee has prebiotic properties in vivo, which contribute to preventing obesity and lifestyle-related diseases [89,90]. A clinical study found a lower risk of advanced prostate cancer associated with higher intakes of caffeic acid and ferulic acid [91].

**Tannins** represent in plants a class of polyphenolic biomolecules with a high molecular weight (500 Da to 20 kDa) [92]. Tannins provide several benefits to plants. They act as a natural defense mechanism against herbivores, insects, and pathogens, deterring them from feeding on the plant. Tannins also play a role in regulating the plant’s growth and development, as well as helping to preserve the structural integrity of plant tissues [92,93]. The most common sources of tannins are bark, stems, roots, leaves, buds, seeds, as well as fruits and vegetables such as grapes, blackberries, strawberries, walnuts, cashew nuts, hazelnuts, mangoes, and tea. Tannins can be divided into three categories: hydrolyzable tannins, condensed tannins (also known as non-hydrolyzable tannins) and phlorotannins [94,95]. Hydrolyzable tannins are complex molecules that can be broken down by hydrolysis into smaller phenolic compounds [96]. A hydrolyzable tannin (e.g., chestnut wood extract) contains repeating structures of gallic (gallotannins) or ellagic (ellagitannins) acid with a sugar core [97]. One of the simplest hydrolyzable tannins is tannic acid, a mixture of digallic acid esters of glucose. Various applications of hydrolyzable tannins are possible, from human/animal nutrition to industrial processing [98]. As an example of its use in animal nutrition, it is well known that dietary supplementation with chestnut wood extract can improve broiler growth performance, nutrient digestibility, antioxidant status, immune response, and lipid metabolism [94,95]. 

Condensed tannins (e.g., mimosa, quebracho, pine, mangrove, and hemlock), also known as non-hydrolyzable tannins, are mainly composed of catechins and anthocyanidin aglycone scaffolds. Condensed tannins dominate the world market with more than 90% of the total commercial tannins, whereas hydrolyzable tannins are limited in nature [94,95]. Unlike hydrolyzable tannins, they cannot be broken down by hydrolysis. Plants use condensed tannins for protection against herbivores and pathogens, as they bind to proteins and prevent them from being digested [99,100]. Aside from contributing to the astringent taste of certain fruits and beverages, condensed tannins also possess antioxidant and anti-inflammatory properties [101]. 

In terms of phlorotannins, they are oligomers of phloroglucinol, a compound found in algae. Phlorotannins have the ability to bind heavy metals, such as cadmium and lead, thereby reducing environmental pollution [102,103].

**Stilbenes** are plant secondary metabolites derived from the phenylpropanoid pathway, associated with plant defense. These compounds can be found in several foods, including berries, grapes, and peanuts, and feature a distinctive structure consisting of two aromatic rings linked by an ethylene molecule. Resveratrol (cis and trans), found in high concentrations in the fresh skin of red grapes, is the main representative of stilbenes. Stilbenes’ health benefits include cardiovascular, anti-obesity, antidiabetic, chemopreventive, and neuroprotective effects [104,105].

**Lignans** are found in the seeds of oleaginous plants (such as flax seeds, sesame seeds, linseeds, and sunflower seeds). However, fibrous plants like rye, whole wheat, vegetables, and fruits also contain lignans, although in smaller amounts. Deglycosylation and demethylation of dietary lignans by the gut microbiota produce human lignan agents such as enterodiol and enterolactone. In recent years, many studies have pointed to the potential therapeutic properties of lignans and their derivatives in cancer chemotherapy and neurodegenerative diseases [105,106]. In terms of health benefit, lignans regulate cholesterol, prevent microbial infections, protect against cancer, improve athletic performance, and reduce inflammation [107,108,109,110,111]. Of note, flaxseed lignans (matairesinol, lariciresinol, and pinoresinol), which are mammalian estrogen precursors, are converted by anaerobic gut bacteria into enterolignans, enterodiol, and enterolactone [112]. As lignan metabolites bind to estrogen receptors, they affect estrogen function, reducing their circulation in the bloodstream and their biological activity, thereby reducing breast cancer risk [113,114]. Of note, excessive flaxseed consumption during pregnancy and breastfeeding can potentially lead to hormonal imbalances due to the high levels of phytoestrogens present in flaxseeds [115].

## 5. Polyphenol-Rich Foods and Their Role in Oxidative Stress Inhibition 

**Figure 4:**
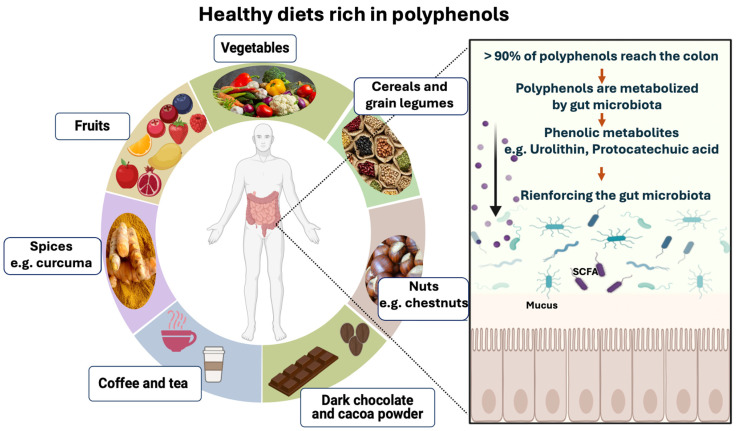
An overview of polyphenol-rich foods and their potential effects on human health in association with gut microbiota. Polyphenols are compounds found in a wide variety of foods, including vegetables (e.g., artichoke heart, parsley, broccoli, celery, onion, garlic, lettuce, leek, zucchini, green bell pepper, tomato, cauliflower, etc.), fruits (e.g., blueberries, strawberries, raspberries, blackberries, cranberries, grapes, cherries, apricots, apples, pomegranates, oranges, grapefruits, etc.), cereals (e.g., wheat, rice, corn, rye, oat, etc.), grain legumes (e.g., beans, chickpeas, lentils, etc.), nuts (e.g., chestnuts, hazelnuts, pecan nuts, almonds, etc.), tea, coffee, dark chocolate, cocoa powder, and spices (e.g., curcuma or turmeric). For IBD patients in remission, it is important to reintroduce gradually restricted foods and drinks (vegetables, fruits, cereals, grain legumes, etc.) that contribute to a balanced diet in line with the “Eatwell Guide”. Polyphenols are mostly complex structures. Approximately 5–10% of food polyphenols are absorbed by the small intestine, while the majority (90–95%) reach the colon. They are then metabolized by the gut microbiota into absorbable simple phenolic compounds (e.g., urolithin, protocatechuic acid, etc.). Dietary phenolic compounds and their aromatic metabolites reinforce the gut microbiota through their prebiotic properties.

Several factors influence polyphenol-rich food absorption in the small intestine, including structural complexity and polymerization [116]. It is estimated that the small intestine absorbs less than 5–10% of ingested polyphenols (Figure 4). Leftover polyphenols (90–95%) can accumulate in the large intestine [116]. Then, the gut microbiota breaks down polyphenol glycosides to form aglycones through the opening of heterocycle rings. This catabolic process reduces polyphenol’s complex structure into low-molecular-weight phenolic metabolites that can be assimilated by gut cells [117,118]. Dietary phenolic substances, along with their aromatic metabolites, have the potential to enhance the gut microbiota community composition through their prebiotic effects (Figure 4) [117,118].

**Figure 5:**
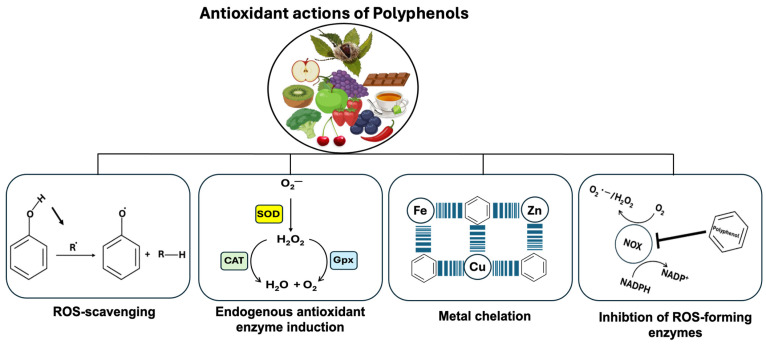
Polyphenols‘ antioxidant properties are mediated by four major mechanisms. Polyphenols can remove ROS directly, while a second action can be attributed to their ability to increase endogenous antioxidant-synthesizing enzymes. Catalase (CAT), glutathione peroxidase (GPx), and superoxide dismutase (SOD) are the most important antioxidant enzymes in cells. SOD and CAT are enzymes that catalyze the breakdown of superoxide and hydrogen peroxide, while GPx is an enzyme that catalyzes the reduction of hydrogen peroxide. As a third action, polyphenols prevent ROS formation by directly acting as metal ion chelators against transition metal ions such as copper (Cu), zinc (Zn), and iron (Fe) that play a critical role in the progression of different diseases including Alzheimer’s disease. For the fourth action, the polyphenol inhibits several enzymes involved in ROS formation (iNOS, NOX, and LOX). For example, the superoxide anion radical (O_2_**^.^**^−^) is produced by members of the NADPH oxidase (NOX) enzyme family, while resveratrol treatment inhibits Nox1 expression and ROS production in macrophages.

Polyphenols’ antioxidant properties are mediated by four major mechanisms (Figure 5). The first polyphenol action involves removing ROS directly, whereas the second increases endogenous antioxidant-synthesizing enzymes (Figure 5). As a third action, polyphenols prevent ROS formation by directly acting as metal ion chelators against transition metal ions such as copper (Cu), zinc (Zn), and iron (Fe) that play a critical role in the progression of different diseases including Alzheimer’s disease. For the fourth action, the polyphenol inhibits several enzymes involved in ROS formation (iNOS, NOX, and LOX). For example, NOX enzymes produce superoxide anion radicals (O_2_**^.^**^−^), while resveratrol treatment of macrophages inhibits LPS-induced Nox1 expression and ROS production [119].

Whenever the amount of ROS produced by cells increases, the cellular defense mechanism against oxidative stress is activated to clear the cells of ROS [120]. In terms of endogenous antioxidant enzyme activity, several enzymes are involved in the cellular antioxidant defense system, including CAT, SOD, GPx, peroxiredoxins (Prxs), and NADPH ubiquinone oxidoreductase (NQO1) [121]. As well as antioxidant enzymes, there are non-enzymatic compounds that reduce ROS, such as glutathione (GSH), ascorbic acid (vitamin C), and tocopherol (vitamin E). During oxidative stress, SODs convert O_2_**^.^**^−^ to H_2_O_2_ which is then eliminated by Prxs, GPx, and CAT. In this case, H_2_O_2_ is converted into H_2_O and O_2_ by CAT to protect cells against oxidative stress [122]. 

## 6. Impact of Polyphenol-Rich Foods on Opportunistic Yeast *Candida albicans* Growth Inhibition

**Figure 6:**
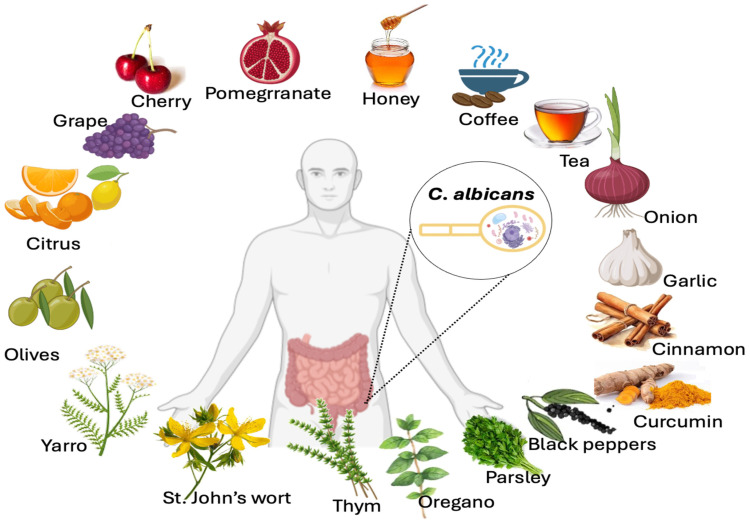
Effect of some representative polyphenol-rich foods on *C. albicans* growth inhibition. Food and spices rich in polyphenols that inhibit *C. albicans* growth include curcumin (curcuminoids), black pepper (p-hydroxybenzoic acid), cinnamon (proanthocyanidins), oolong tea or green tea (e.g., catechins), yarrow, St. John’s wort, oregano, thyme (carvacrol and thymol), coffee (phenolic acids), parsley (flavonoids), garlic (phenolic acids and flavonoids), onions (anthocyanins and flavonols), honey (flavonoids), cherries (procyanidins and quercetin), grapes (resveratrol), citrus fruits (naringin), pomegranates (caffeic acid, gallic acid, and epigallocatechin), and olives (hydroxytyrosol).

In addition to polyphenols’ properties in preventing ROS generation, maintaining intestinal homeostasis, and boosting the biodiversity and health of the microbiota, they have also been shown to contribute to the elimination of pathogens, such as *Candida albicans* (Figure 6). This yeast is an opportunistic fungus that lives in humans’ vaginal and intestinal tracts [123,124,125]. This microorganism is the most common cause of yeast infections in humans. *C. albicans* infections can range from superficial to systemic and life-threatening [125]. Different factors may facilitate *C. albicans* overgrowth and its migration from the gut to the vital organs. These factors include changes in the gut microbiota, epithelial barrier ruptures, and immune system dysfunction [124]. Another important aspect is the fact that *C. albicans* overgrowth is associated with inflammatory diseases [126,127,128]. Evidence from experiments and clinical studies suggests a link between *C. albicans* and CD [129,130]. Of note, unhealthy diets high in fats and sugars, particularly processed foods consumed in Western countries, affect the gut microbiota, contributing to intestinal inflammation and fungal overgrowth [68,131]. The antifungal properties of polyphenol-rich foods have attracted considerable attention in recent years, especially those belonging to the flavonoid class (flavanols and flavonols) [132,133,134]. These polyphenols inhibit virulence factors such as biofilm formation, *C. albicans* filamentation, modulation of the fungal cell wall, and reduction of fungal adhesion to host cells [132,133,134].

Food and spices rich in polyphenols that inhibit *C. albicans* growth include curcumin (curcuminoids), black pepper (p-hydroxybenzoic acid), cinnamon (proanthocyanidins), oolong tea or green tea (e.g., catechins), yarrow, St. John’s wort, winter savory, or willow gentian, thyme (carvacrol and thymol), coffee (phenolic acids), parsley (flavonoids), garlic (phenolic acids and flavonoids), onions (anthocyanins and flavonols), honey (flavonoids), cherries (procyanidins and quercetin), grapes (resveratrol), citrus fruits (naringin), pomegranates (caffeic acid, gallic acid, and epigallocatechin), and olives (hydroxytyrosol).

**Curcumin**, curcuminoids are one of the main compounds found in *Curcuma longa* rhizome (turmeric). Gut microbiota is directly regulated by curcumin, and curcumin itself is bio-transformed into active metabolites by gut microbiota [135]. In terms of curcumin’s role in fungal growth inhibition, the study of Shahzad et al. showed that curcumin inhibits the expression of hwp1 and als3 that affect *C. albicans* biofilm formation [136]. 

**Black pepper** contains piperine, a bioavailability enhancer, which increases curcumin’s bioavailability by 2000% [137]. One of the most prominent phenolic acids in black pepper is p-hydroxybenzoic acid [138]. Of note, piperine demonstrated concentration-dependent antibiofilm activity of *C. albicans* [139].

**Cinnamon**, commercial cinnamon contains the polyphenol proanthocyanidins, which may contribute to the health benefits [140]. A study conducted by Atai et al. found that *Absinthium artemisia*, eucalyptus, onion, cinnamon, curcumin, sage, mint, and *Calendula officinalis* all showed antifungal activity against *C. albicans* strains. Cinnamon was more potent and effective than onion, mint, *C. officinalis*, and sage. In contrast, curcumin, *A. artemisia*, and eucalyptus showed similar antifungal effects [141].

**Tea**, the antioxidant activity of black tea is lower than that of oolong teas or green teas as it contains the lowest level of polyphenols (catechins) [142]. Additionally, green tea was shown to have the highest antimicrobial properties, particularly against *C. albicans* compared to other types of tea [142]. It has been shown that catechins and flavins in black tea are both anti-*Candida* agents and inhibit all *Candida* species tested with the lowest MIC of 6.25 µg/mL observed for *C. albicans* (Figure 6) [143]. These catechins and flavins in black tea cause cell wall damage to *C. albicans* [143].

Many medicinal plants, traditionally used to treat digestive problems, have been shown to suppress pathogen growth, including *C. albicans*, and to promote the growth of probiotic bacteria, such as **yarrow**, **St. John’s wort**, **winter savory, or willow gentian** extracts [144]. Additionally, carvacrol and thymol from **thyme and oregano** both exhibit strong fungicidal properties against all *Candida* isolates through inhibition of ergosterol biosynthesis as well as disruption of the integrity of the membrane of *C. albicans* [145]. Thymol is not only effective in inhibiting *C. albicans* adhesion to host cells, but it has also been shown to be effective in preventing fungal infections in host cells [146,147].

**Coffee,** having the remarkable contents of phenolic acids, represents one of the most consumed beverages and is also a major contributor to dietary antioxidant intake [148]. A significant reduction in the content of ergosterol, chitin, and β-glucan in *Candida* species was observed following treatment with spent coffee grounds, suggesting that these extracts target the synthesis of membranes and cell walls of *Candida* species [149].

**Parsley,** due to its high polyphenol content, especially of flavonoids, demonstrates antibacterial properties [133]. A study conducted by Arismunandar et al. showed that parsley (*Petroselinum crispum*) extract inhibits *C. albicans* growth [133].

**Garlic,** many phytomolecules found in garlic are biologically active, including organosulfur compounds, phenolic acids, allyl thiosulfinates, flavonoids, and vitamins [150]. Through its antioxidant activities (phenolic acids and flavonoids), garlic protects against ROS generation in the body. Additionally, allicin, allyl cysteine, allyl disulfide, and alliin are the four main compounds constituting garlic compounds that scavenge free radicals. Inhibition of succinate dehydrogenase is one mechanism by which allicin inhibits fungi growth [151]. Garlic has been reported to affect the lipid composition of the outer surface of *C. albicans* [152]. Furthermore, garlic extract inhibits *C. albicans* growth by forming pits on its surface [151].

**Onions**, anthocyanins and flavonols are the main flavonoid classes found in onions. Some onion varieties are red due to anthocyanins. Onions have yellow and brown skin due to flavonols such as quercetin. The research of Doddanna et al. showed that onion leaves and bulb extracts inhibit *C. albicans* growth [153]. 

In comparison with onion or honey alone, onion juice extracted from red Egyptian onion reduced the growth of *C. albicans*, while the honey–onion mixture was significantly more effective than onion juice alone [154]. **Honey** polyphenols, specifically flavonoids, have been found to possess natural antifungal properties that effectively combat *C. albicans* by protecting against the yeast-to-hyphal transition that occurs in *C. albicans* [134].

In addition to plants and herbs, fruits also contain polyphenols that play an antifungal role in preventing *C. albicans* by acting as an antifungal [155,156,157]. 

**Cherry** (*Prunus cerasus* L.) is an excellent source of procyanidins and quercetin that attenuate *C. albicans* adherence to the oral cavity epithelium [155]. 

Resveratrol derived from **grapes** displays potent fungicidal activity by significantly increasing intracellular trehalose content in the cells. This trehalose accumulation was induced by stress responses to resveratrol action, and *C. albicans* showed an arrest in their cell-cycle processes at S phase [156].

Naringin, a flavonoid found in **citrus fruits**, exhibits potent antifungal properties due to its ability to disrupt mitochondrial function in *C. albicans*. This disruption ultimately leads to mitochondrial dysfunction and triggers apoptosis in fungal cells [132].

**Pomegranate** (*Punica granatum* L.) juice has high polyphenol content, including caffeic acid, gallic acid, and epigallocatechin [158]. The pomegranate peel contains ellagitannin, flavonoids, triterpenes, and phenols which have been shown to have an antibacterial effect. Pomegranate peel inhibits fungal growth and *C. albicans* biofilm formation [157]. 

Polyphenol-rich Mediterranean fruits include **olives** (*Olea europaea*). Hydroxytyrosol is the main phenolic component in olives that contributes to their health benefits [159]. Hydroxytyrosol acts as a radical scavenger and induces apoptosis in cancer cells. Additionally, hydroxytyrosol exhibits antimicrobial properties [160]. The study conducted by Zoric et al. showed that hydroxytyrosol inhibits the transition of unicellular *C. albicans* yeast into filamentous forms and induces changes in the hydrophobicity of cell surfaces, factors affecting *C. albicans* adhesion to cell hosts [161]. 

## 7. Conclusions

In summary, the production of ROS is increased during inflammation by immune cells, such as monocytes and neutrophils, which causes further tissue damage. Oxidative stress, associated with increased ROS generation, is a major risk factor for IBD pathogenesis and progression. Additionally, a decrease in antioxidant enzyme expression (CAT, GPx, and SOD) in the colonic mucosa, submucosa, and serosa has been observed in IBD patients.

Currently, industrialized lifestyles expose us to a variety of exogenous factors (smoking, processed food, hydrogenated oils, alcohol, chronic stress, air pollution, heavy metals, and UV light) and endogenous conditions (mitochondria and phagocyte NADPH oxidases) that deteriorate the digestive tract, especially in IBD patients. These unhealthy factors can trigger chronic inflammation and oxidative stress, which further exacerbate IBD pathogenesis and progression.

Many alternative therapeutic strategies have been developed against oxidative stress along with conventional therapy to alleviate IBD pathogenesis. Some alternative therapeutic strategies that reduce oxidative stress in IBD include antioxidants, such as LOX, NOX inhibitors and melatonin. Furthermore, healthy lifestyle changes, such as adopting a healthy diet rich in fruits and vegetables as well as medicinal plants abundant in β-carotene (pro-vitamin A), vitamin C, vitamin E, minerals, omega-3 polyunsaturated fatty acids, and polyphenols. Additionally, regular exercise combined with food rich in probiotics and prebiotics can reduce oxidative stress and inflammation. Of note, the natural presence of antioxidants (bioactive peptides) and lactoferrin in some foods (e.g., camel milk) provides a potential opportunity for protection against oxidative stress in IBD patients.

Polyphenol-rich foods have attracted growing interest from scientists due to their antioxidant properties. Polyphenols are natural compounds found in plants, fruits, vegetables, and nuts. These polyphenol-rich foods exhibit antioxidant properties and protect the body from oxidative damage caused by free radicals. Polyphenols, particularly those belonging to the flavonoid class (flavanols and flavonols), have been shown to possess antifungal properties, particularly against *C. albicans*. Polyphenol-rich foods that inhibit *C. albicans* growth include curcumin, black pepper, cinnamon, oolong tea or green tea (e.g., catechins), yarrow, St. John’s wort, winter savory, or willow gentian, thyme (carvacrol and thymol), coffee, parsley, garlic, onions (anthocyanins and flavonols), honey (flavonoids), cherries, grapes (resveratrol), citrus fruits (naringin), pomegranates (caffeic acid, gallic acid, and epigallocatechin), and olives (hydroxytyrosol). These polyphenols inhibit virulence factors such as biofilm formation, *C. albicans* filamentation, modulation of the fungal cell wall, and reduce fungal adhesion to cell host.

Overall, a healthy and balanced diet abundant in polyphenols along with regular physical activity and practicing stress management techniques and avoiding the industrialized lifestyles of our modern society (the factors listed above) can minimize oxidative stress damage and prevent infectious diseases. Various factors influence polyphenol absorption in the gut, including polyphenol type, dietary components, gut microbiota composition, and individual gut health differences. Identification of these factors may enable us to develop strategies for improving polyphenol gut absorption and maximizing their health benefits.
